# Development of Azo Dye Immobilized Sulfonated Poly (Glycidyl Methacrylate) Polymer Composite as Novel Adsorbents for Water Treatment Applications: Methylene Blue Immobilization Isotherm, Kinetic, Thermodynamic, and Simulations Studies

**DOI:** 10.3390/molecules27238418

**Published:** 2022-12-01

**Authors:** Mohamed R. El-Aassar, Tamer M. Tamer, Mohamed Y. El-Sayed, Ahmed M. Omer, Ibrahim O. Althobaiti, Mohamed E. Youssef, Rawan F. Alolaimi, Emam F. El-Agammy, Manar S. Alruwaili, Omar O. Rabhy, Mohamed S. Mohy-Eldin

**Affiliations:** 1 Chemistry Department, College of Science, Jouf University, Sakaka 2014, Saudi Arabia; 2 Polymer Materials Research Department, Advanced Technology and New Materials Research Institute, (ATNMRI), City of Scientific Research and Technological Applications (SRTA-City), New Borg El-Arab City, Alexandria 21934, Egypt; 3 Chemistry Department, College of Science and Arts, Jouf University, Gurayat 77217, Saudi Arabia; 4 Computer Based Engineering Applications, Informatic Research Institute (IRI), SRTA-City, New Boarg El-Arab City, Alexandria 21934, Egypt; 5 Physics Department, College of Science, Jouf University, Sakaka 2014, Saudi Arabia; 6 School of Engineering and Information Technology, University of New South Wales, Canberra, ACT 2600, Australia

**Keywords:** composite, MB, sulphonated poly(glycidyl methacrylate), adsorbent, water treatment, adsorption, isotherm, kinetic, thermodynamic, stimulation

## Abstract

Methylene blue (MB) immobilized onto a sulfonated poly(glycidyl methacrylate) (SPGMA) polymer composite has been developed as a novel adsorbent for water treatment applications. The MB adsorptions onto sulfonated poly(glycidyl methacrylate) polymer characters have been studied. The adsorption isotherms, namely Langmuir and Freundlich, have been investigated. Other isotherm models. As a compromise between the Freundlich and Langmuir isotherm models, such as the D–R isotherm and the Temkin isotherm, have been compared. The results indicated that the adsorption process followed the Freundlich isotherm model, indicating heterogeneous surface site energies and multi-layer levels of sorption. This study selected three linear kinetic models, namely pseudo-first order, pseudo-second order, and Elovich, to describe the MB sorption process using SPGMA negatively charged nanoparticles (430 nm). The obtained data revealed that the adsorption process obeyed the pseudo-second-order kinetic model, suggesting that the rate-limiting step in these sorption processes may be chemisorption. Furthermore, the thermodynamic parameters have been evaluated. Moreover, the interaction of the MB molecules with SPGMA nanoparticles has been simulated using the governing equation that describes ion exchange resin derived from Nernst—Plank equations between two ion species. Finally, the developed MB-SPGMA composite adsorbent (27 mg/g) wastested for the first time for the removal of Cr^6+^ ions and Mn^7+^ metal ions from dichromate and permanganate-contaminated waters under mild adsorption conditions, opening a new field of multiuse of the same adsorbent in the removal of more than one contaminant.

## 1. Introduction

Ionic and non-ionic dyes are used in a variety of sectors, including food, paper, textiles, and carpet. As a result, dyes are polluting the wastewater produced by these enterprises. Around the world, 10,000 tonnes are used each year, and at least 1000 tonnes are emitted in the wastewater from these industries [1,2].

The most dangerous source of dyes is thought to be textile dyes [3,4]. The release of colors into the water system has a negative effect on aquatic life directly as well as indirectly on human life. Cationic dyes such asmethylene blue (MB) are frequently utilized as coloring agents around the world [5]. Therefore, corrective action is required to remove dyes from wastewater. Physical, chemical, and even biological approaches have all been explored to remove colors from wastewater [6]. Physical and chemical procedures have emerged as the most problematic among them from a number of perspectives, including the ease of design, accessibility, and the capacity to handle dyes more concentrated than with other techniques [7,8,9,10]. The search for alternative adsorbents has been fueled by studies into their viability and affordability over the past few decades. Regardingthe removal of various dyes, numerous publications have been published [11,12,13]. Among other methodologies, the physiochemical methodology is well regarded [14,15,16,17,18,19,20,21]. The key interaction in the removal process is the physical adsorption of the dyes or chemicals attached to the surface of the adsorbent surface by electron exchange [22]. The effectiveness of adsorption is impacted by various factors. Some of these have to do with the adsorbent’s structure, while others have to do with the operational circumstances [23].

Based on a variety of factors, including the initial cost, convenience of design, simplicity of operation, and insensitivity to harmful compounds, the adsorption technique is typically the most preferred in the contaminate removal process [7,8,9,10,11,12,13,14,15,16,17,18,19,20,21,22,23,24]. Activated carbon has demonstrated a wide range of applications in this setting [25]. However, other polymer-based adsorbents have been studied for the removal of Methylene Blue dye from contaminated wastewater, including grafted cotton textiles [26], carboxylated alginate beads [27], and pyrazole-g-poly(glycidyl methacrylate) [28]. Additionally, various adsorbents, particularly those based on nanopolymers, have been used [29,30,31,32]. Polystyrene was created by Mohy-Eldin et al. as a nanoparticle ion exchange resin for use in water purification applications. Polystyrene and poly(4-vinylbenzenesulfonic acid) copolymer nanoparticles were created using the sedimentation polymerization method, and the ion exchange mechanism was modelled [33].

Additionally, as the industry has developed, heavy metal pollution of the environment has spread to every country. The most common harmful heavy metals utilized and found in the environment includemany heavy metals, such as nickel, copper, cadmium, manganese, and chromium [34,35]. Low concentrations of certain metals are necessary as co-factors for enzymes, whereas high concentrations are hazardous to live cells because they obstruct metabolism. Bioremediation has received a great dealof attention recently due to its significant potential for purifying the ground of heavy metal contamination [36].

As a powerful oxidizing agent for the oxidative treatment of several organic and inorganic chemicals in soil and water solutions, potassium permanganate is frequently utilized in multidisciplinary processes [37,38]. Few papers have, to our knowledge, discussed the elimination of permanganate ions. Activated orange peel powder [39], activated carbon [40,41], Prosopis cineraria leaf powder [42], and millet husk [43] are some of the different adsorbents that have been used to remove Mn^7+^ from wastewater. Adsorption is thought to be a cheap and effective way for this task.

Chromium can primarily be found in the natural world as Cr^6+^ or Cr^3+^. In contrast to Cr^6+^ species, which are highly soluble and mobile in aqueous solutions, Cr^3+^ species are less soluble and more stable [44]. Because Cr^6+^ is more mobile than Cr^3+^, it has a greater potential to contaminate groundwater. The high reactivity and probable carcinogenicity of Cr^6+^ are connected toits high risk [45]. Acute exposure to Cr^6+^ can result in respiratory issues, dermatitis, internal bleeding, nausea, diarrhea, liver, and kidney damage [5]. Acute toxicity, irritation, nasal septum ulceration, and respiratory sensitization (asthma) can all be brought on by inhalation [46]. Liver and kidney functioning may be impacted by consumption. Skin contact has the potential to cause serious burns and systemic poisoning and impede the healing of cuts and scrapes. This could result in severe chronic allergic contact dermatitis and ulceration if not addressed right away. Exposure to the eyes could harm them permanently. The removal of Cr^6+^ from wastewater using various adsorbents, including charcoal [47], activated carbon from various sources [48,49,50], polyaniline and its composites [51], and chitosan [52], is also thought to be a cheap and effective method. Diazo acid Blue 11 (AB 113) was created by Nicoleta Mirela Marin [53] and used as a chelating agent in natural and acrylic polymers to selectively remove Zn^2+^, Mn^2+^, and Cr^3+^ from acid-polluted wastewater.

The adsorption of heavy metals, noble metals, and dyes in wastewater has recently attracted a great dealof attention and has been successfully applied using poly(glycidyl methacrylate) (PGMA)-based resins [54]. PGMA-based resins have good mechanical strength, high tensile strength, acidic and alkaline resistance, and wear resistance [55]. The most notable features of PGMA include its porous structure, the presence of highly reactive epoxy groups, and the ease with which it can be functionalized with diverse groups by straightforward chemical reactions [56]. Younis et al. [57] created an amined poly(glycidyl methacrylate) nanosorbent for the treatment of wastewater that was contaminated with phenol and malathion. Aversa et al. [58] investigated how the modified glycidyl methacrylate polymer exchange group affected phenol removal in batch and continuous-flow methods. Using grafted Polypyrrole chains, Yu et al. [59] created magnetic Poly(glycidyl methacrylate) microspheres for the high-capacity removal of Congo red dye from aqueous solutions. Magnetic Poly(glycidyl methacrylate) resin was created by Chen et al. [60] for the treatment of drinking water. To purify glucosinolates from cruciferous vegetables, Cheng et al. [61] created Poly(glycidyl methacrylate) (PGMA) and amine-modified PGMA adsorbents. Benaglia et al. [62] described the post-polymerization processes used to create a range of polymers with different properties from poly(glycidyl methacrylate) (PGMA) produced using RAFT. Waly et al. [63] created an adsorbent for the removal of the dyes C.I. Acid Black 194 and C.I. Reactive Black 5 from wastewater using an amino-functionalized cellulose-poly(glycidyl methacrylate) graft copolymer (AM-Cell-g-PGMA). Sulfonated Poly(glycidyl methacrylate) nanoparticles were created by Mohy-Eldin colleagues [29,63] for the removal of Cadmium ions from contaminated water. Additionally, they created sulfonated PGMA-g-cellophane membranes [64] and sulfonated PGMA-g-Nafion membranes [65] for use as ionic conducting membranes in fuel cells.

For the first time, a novel adsorbent for water treatment applications has been created in the first section of our recently published work by immobilizing methylene blue (MB) as the first pollutant onto composites of sulfonated poly(glycidyl methacrylate) (SPGMA) polymers through an adsorption technique. The elimination of metal ions such Cr^6+^ and Mn^7+^ from dichromate and permanganate-contaminated water, as the second contaminant, has been further studied using the created MB-SPGMA composite [66].

Adsorption isotherms such as Langmuir and Freundlich, as well as other isotherm models that serve as a compromise between the Freundlich and Langmuir isotherm models such as the D-R isotherm and the Temkin isotherm, have been used in the current study to analyze the first step of the MB immobilization by adsorption onto sulfonated poly(glycidyl methacrylate) polymers. In this study, three linear kinetic models, pseudo-first order, pseudo-second order, and Elovich, were chosen to describe how MB sorption occurs when SPGMA particles are used. The thermodynamic parameters have also been assessed. Last but not least, the governing equation that describes ion exchange resin and is derived from Nernst–Plank equations between two ion species has been used to simulate the interaction of the MB molecules with SPGMA particles. As a model of toxic metal ions, Cr^6+^ and Mn^7+^ ions were tested for removal from dichromate- and permanganate-contaminated waters using the newly developed MB-SPGMA composite adsorbent under mild adsorption conditions, opening a new field of harmful anion removal from contaminated water.

## 2. Results and Discussion

The development of the MB-SPGMA adsorbent using the adsorption process of MB molecules onto SPGMA nanoparticles has been characterized using isotherm, kinetic, thermodynamic, and simulation models as follows. Moreover, the developed MB-SPGMA adsorbent has been examined in removing harmful ions from contaminated water.

### 2.1. Methylene Blue Concentration and Adsorption Isotherms

**Figure 1:**
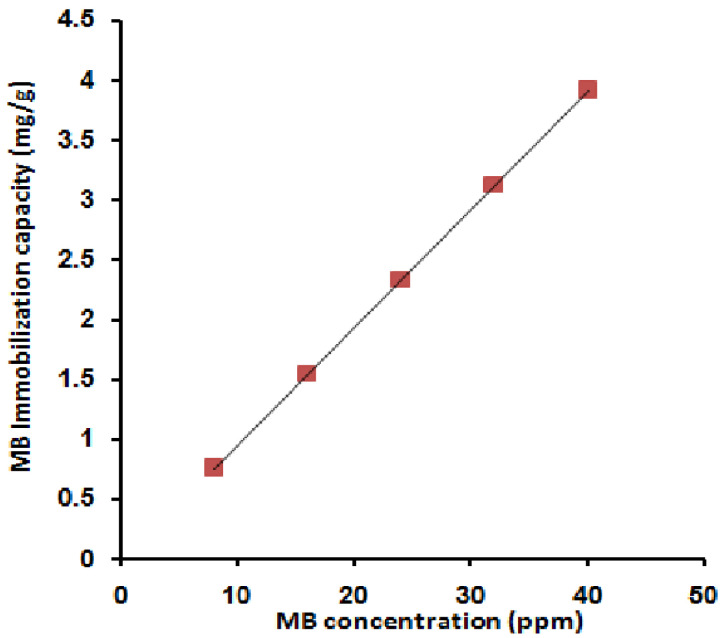
Effect of MB concentration on its immobilization capacity; [MB 10 mL, room temperature, 30 min, 0.1 g SPGMA, rpm 200, and pH 6.5].

Figure 1 shows the effect of variation of the MB concentration on the adsorption capacity. The effect of the initial dye concentration factor depends on the immediate relation between the dye concentration and the available binding sites on an adsorbent surface [12]. From the figure, the increase in the initial dye concentration causes a linear increase in the loading capacity of the adsorbent, and this may be due to the high driving force for mass at a high initial dye concentration [67]. That indicates a high number of active sites relative to the number of MB molecules in the liquid phase of all the MB concentrations used where free active sites are still available. This postulation has been confirmed by the linear increase in the adsorption capacity to reach the highest value of 3.94 mg/g.

Freundlich and Langmuir isotherm models are the most common isotherm models used in almost all the publications that deal with the characterization of the adsorption process. Unfortunately, the two models are opposite each other.

**Figure 2:**
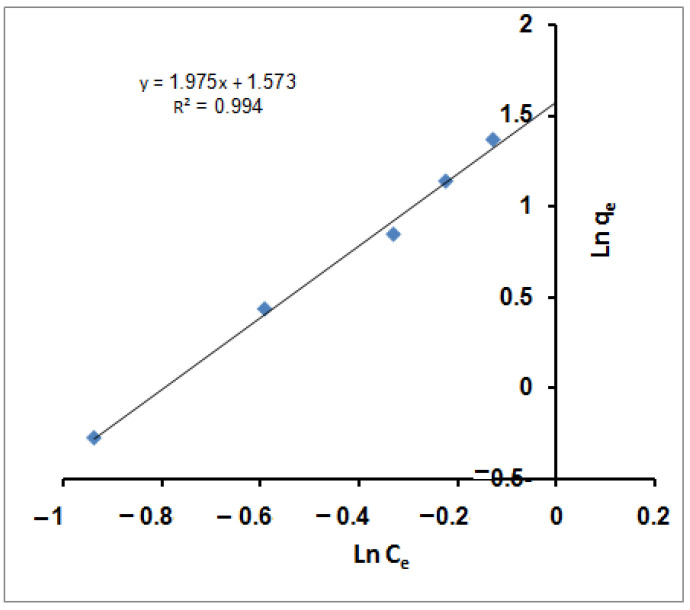
Freundlich isotherm for MB adsorption using SPGMA.

The Freundlich isotherm is a widely used equilibrium isotherm model but provides no information on the monolayer sorption capacity, in contrast to the Langmuir model [68,69]. The Freundlich isotherm model is the first used isotherm model, which postulates heterogeneous surface site energies and multi-layer levels of sorption. The linear mathematical formula of the model is expressed as the following equation [70]:

ln q_e_ = ln K_F_ + (1/n*_f_*) ln C_e_ (1)

q_e_ (mg/g) and C_e_ (mg/L) represent the adsorbent capacity and the adsorbate ions concentration at equilibrium. The indicators of the adsorption capacity and adsorption intensity are given by K_F_ and n*_f_* Freundlich constants. Linear fits of the sorption data of MB molecules are provided in Figure 2. According to the figure, the Freundlich equation predicts that the MB molecules concentration on the sorbents will increase as long as there is an increase in the MB molecules’ concentration; this is compatible with the experimental results. Furthermore, the correlation coefficient (R^2^) value (0.994) demonstrated that the removal of MB molecules obeyed the Freundlich isotherm. The values of Freundlich constants n*_f_* (0.506) and K_F_ (4.822) are estimated from the slope and intercept of the linear plot. From the assessed value of n*_f_*, it was found that n*_f_* ˂ 1 dictated non-favorable sorption for MB molecules with the SPGMA particles [71].

On the other hand, the Langmuir isotherm assumes a completely homogeneous surface with a finite number of identical sites and little interaction between adsorbed molecules, which results in monolayer sorption. The linear mathematical formula of the model is presented by the following equation [72]:

(C_e_/q_e_) = (1/q_m_K) + (C_e_/q_m_) (2)

q_m_ is the maximum monolayer adsorption capacity (mg/g) and K is the adsorption energy (L/mg).

**Figure 3:**
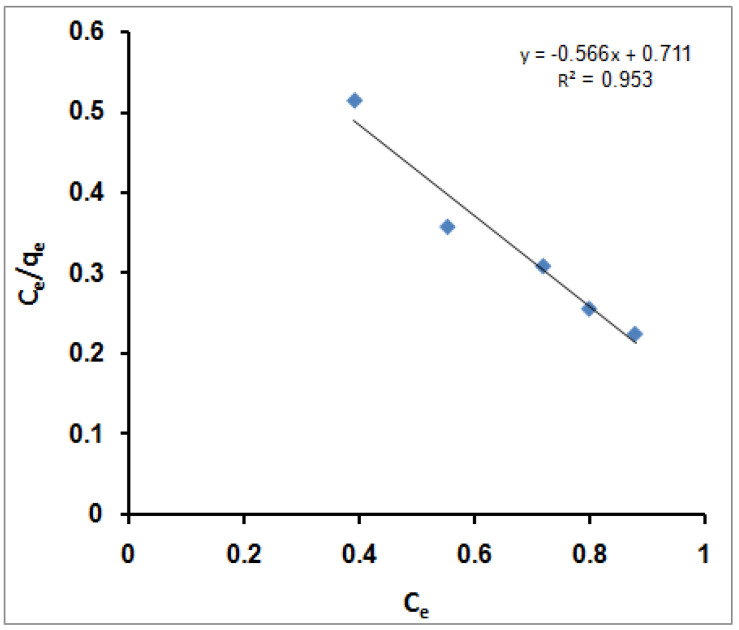
Langmuir isotherm for MB adsorption using SPGMA.

A plot of (C_e_/q_e_) versus C_e_ should present a straight line of the slope (1/q_m_) and intercept (1/q_m_K). Figure 3 illustrates a linear plot of the Langmuir equation for MB molecules immobilization onto SPGMA polymer at various initial MB molecules concentrations. According to the R^2^ value, the Langmuir equation does not represent the sorption process of MB molecules very well; the R^2^ value is 0.953. That indicates an excellent mathematical fit. Furthermore, it was found that the calculated value of q_m_ (1/slope) is 1.767 mg/g and that of K (intercept/slope) is 1.256 L/mg. That indicates that the SPGMA was highly efficient for MB molecule adsorption and had low-energy sorption (1.256 L/mg), which referred to the affinity of SPGMA towards the MB molecules.

Prediction of the favorable or unfavorable of the adsorption system and the essential characteristics defined by a dimensionless separation factor (R_L_) are used and calculated according to the following equation [73]:

R_L_ = 1/(1 + KC_0_) (3)

C_0_ is the MB molecules’ initial concentration (mg/L). The calculated values of R_L_ for MB molecules’ adsorption (Table 1) show favorable adsorption because the R_L_ values ranged between 0 and 1 [74,75]. That again confirms that the Langmuir isotherm was favorable for the sorption of MB molecules onto SPGMA under the conditions used in this study.

Other isotherm models are a compromise between the Freundlich and Langmuir isotherm models, such as the D-R isotherm and the Temkin isotherm. The D-R isotherm is a derivative from the Langmuir isotherm but is more general and rejects the constant adsorption potential assumption [71]. The D-R isotherm is expressed as follows:

ln q_e_ = ln*V*′_m_ − *K*′Ɛ^2^ (4)

where q_e_ is the amount of MB molecules adsorbed per unit of adsorbent mass (mg/g), *V*′_m_ is the D-R sorption capacity (mg/g), *K*′ is a constant related to the removal energy (mol^2^/kJ^2^), and Ɛ is the Polanyi potential. Ɛ is calculated with the following equation:

Ɛ = *RT* (1 + 1/C_e_) (5)

*R* is the gas constant (8.314 × 10^−3^ kJ/mol K) and *T* is the temperature (K). The constant *K*′ gives the mean free energy of sorption per molecule of the sorbate (*E*) when it is transferred to the surface of the solid from infinity in the solution. This energy provides information about the physical and chemical features of the sorption process [75] and can be calculated using the following equation [76]:

*E* = (2 K′)^−0.5^ (6)

This energy provided information about the sorption mechanism. It was perceived as the amount of energy required to transfer 1 mole of the adsorbate from infinity in the bulk of the solution to the site of sorption. If *E* is between 8 and 20 kJ/mol, the sorption process follows a chemical ion exchange, and if *E* < 8 kJ/mol, the sorption process has a physical nature [77,78].

**Figure 4:**
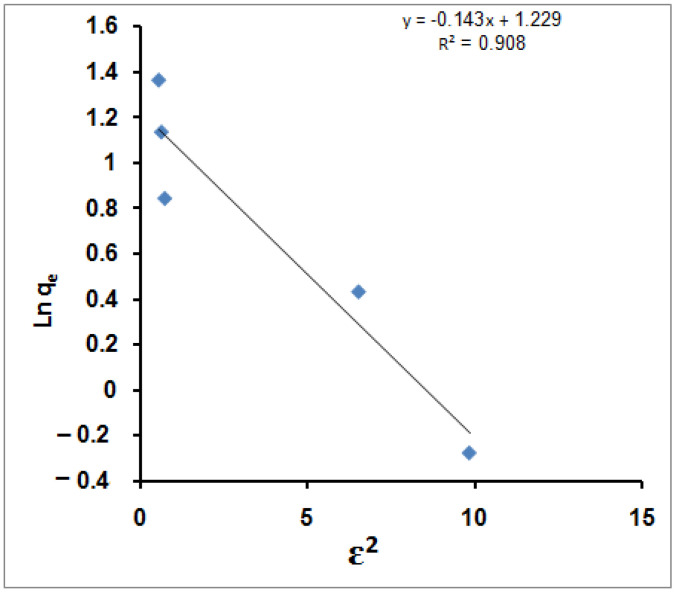
D-R isotherm for MB adsorption using SPGMA.

The D-R isotherm model was applied to the equilibrium data obtained from the empirical studies for MB molecules’ adsorption using SPGMA to determine the nature of the sorption processes (physical or chemical). For example, a plot of ln q_e_ against Ɛ^2^ is given in Figure 4.

The D-R plot yields a straight line with the R^2^ value equal to 0.908, which indicates that the D-R model fits the experimental data less compared with the Langmuir and Freundlich isotherm models. According to the plotted D-R isotherm, the model parameters *V*′_m_, *K*′ and *E* are determined, and their values are 3.4205 mg/g, 0.1435, and 1.867 kJ/mol. The calculated removal energy (*E* < 8 kJ/mol) indicates that the MB molecules’ adsorption processes could be considered physisorption in nature [79]. Therefore, it is possible that physical means such as electrostatic forces played a significant role as sorption mechanisms for the sorption of MB molecules in this work. Furthermore, the adsorption of other metal ions onto different adsorbents has been fitted with a D-R isotherm, for example, natural clinoptilolite tuff modified with hexadecyltrimethyl ammonium bromide (HDTMA) and dithizone (DTZ) in the removal of Pb^2+^ cations [80] and aspartic acid (ASP)-modified clinoptilolite in the removal of Cu^2+^ ions [81].

Finally, the Temkin isotherm considers the impact of indirect adsorbent/adsorbate interactions on the adsorption process, which linearly reduces the heat of the adsorption of all molecules in a layer [82]. That can be expressed in a linear form as follows [83]:

q_e_ = *B* ln *K_T_* + *B* ln C_e_ (7)

**Figure 5:**
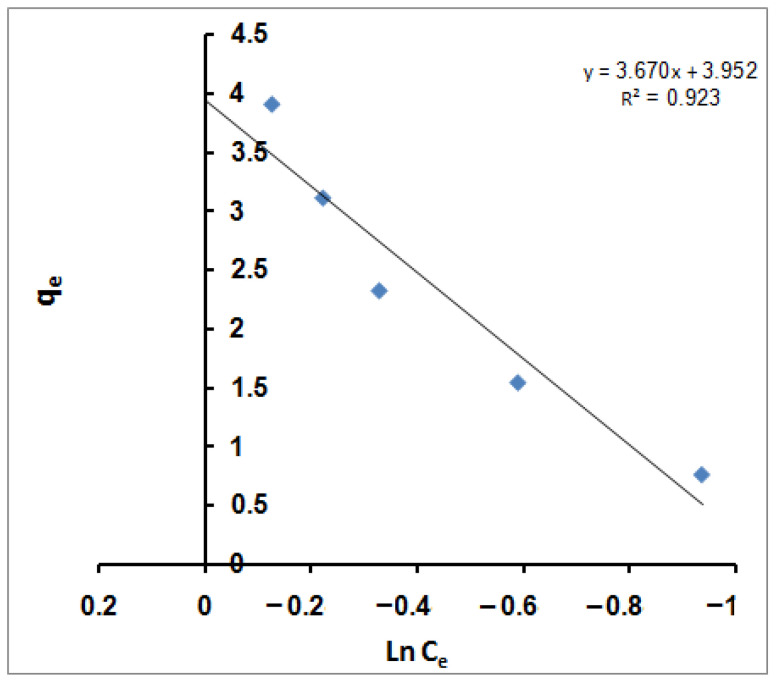
Temkin isotherm for MB adsorption using SPGMA.

*K_T_* is the Temkin equilibrium-binding constant corresponding to the maximum binding energy, and *B* is the Temkin constant related to the heat of sorption. A plot of q_e_ versus ln C_e_ (Figure 5) enables the determination of isotherm constants *B* and *K_T_* from the slope and the intercept, respectively.

The calculated value of *K_T_* is 2.936 L/g, representing the equilibrium-binding constant corresponding to the maximum binding energy; however, constant *B*, which is 3.6704 J/mol, is related to the heat of sorption for the SPGMA matrix.

Finally, all the R^2^ values obtained from the four equilibrium isotherm models applied to MB molecules’ adsorption on SPGMA nanoparticles are summarized. The Freundlich isotherm model yielded the highest R^2^ value (0.994). That showed that MB molecules’ adsorption on the polymer was described well by this model, which considers heterogeneous surfaces site energies and multi-layer levels of sorption. On the other hand, the Langmuir isotherm yielded the next highest R^2^ value (0.954). It assumes an entirely homogeneous surface with a finite number of identical sites and little interaction between adsorbed molecules, which results in monolayer sorption. Finally, the Temkin isotherm and the D–R isotherm, which are a compromise between the Freundlich and Langmuir isotherm models, had lower R^2^ values of 0.923 and 0.908.

In conclusion, all the studied isotherms show excellent fitness of the adsorption results according to the regression coefficient of the obtained lines (R^2^), which ranged between 0.91 and 0.998. This finding indicates the coexistence of monolayer adsorption (Langmuir isotherm) and multilayer adsorption (Freundlich isotherm). The most dominant is the Freundlich isotherm, which has higher fitness of the adsorption data. This explanation is supported by the data obtained in our previous published study, where the PGMA nanoparticles without and with sulfonation removed 100% of the MB from a 10 ppm solution [66]. Based on our previous study of the sulfonation conditions, the resulting SPGMA nanoparticles in our current study are partially sulfonated [27]. In other words, the developed sulfonated PGMA nanoparticles are a copolymer of PGMA and SPGMA. Accordingly, the induced sulfonic ionic sites in the SPGMA region contributed mainly to the immobilization process through the chemosorption step. On the other hand, the PGMA region contributed mainly to the immobilization process through the physiosorption step. This finding explains the D-R isotherm’s lower fitness of the adsorption results, which eliminates the variation of the adsorption potential assumption. The calculated removal energy (*E* < 8 kJ/mol) indicates that the MB molecules’ adsorption processes could be considered physisorption. Therefore, physical means such as electrostatic forces played a significant role as sorption mechanisms for the sorption of MB molecules in this work. Furthermore, the Temkin isotherm considers the impact of indirect adsorbent/adsorbate interactions on the adsorption process, which linearly reduces the heat of adsorption of all molecules in a layer. The obtained results indicate the absence of indirect adsorbent/adsorbate interactions in the adsorption process since the R^2^ value is less than the Freundlich and Langmuir isotherm models and there is an absence of compromise between them.

### 2.2. Methylene Blue Immobilization Time and Adsorption Kinetics

**Figure 6:**
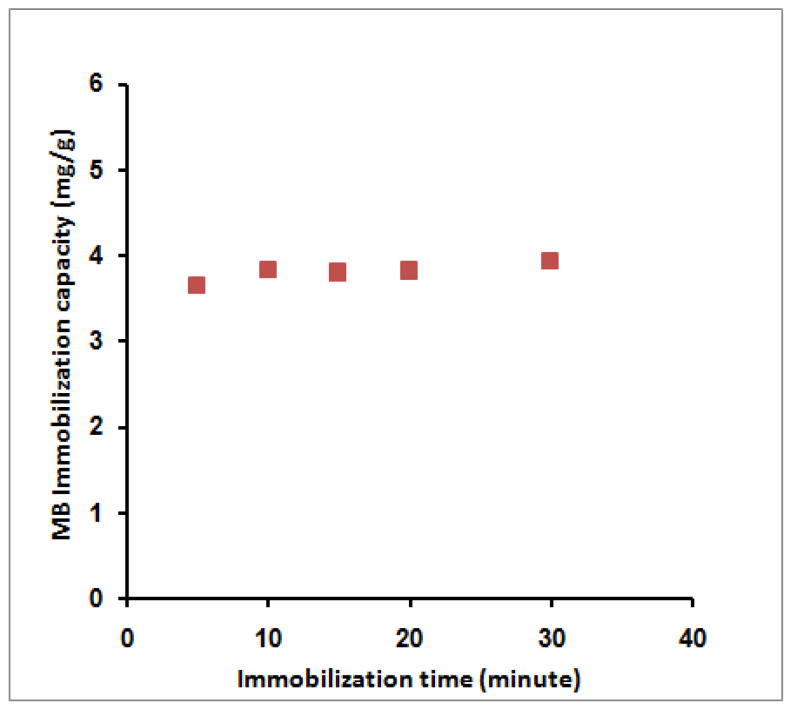
Effect of MB immobilization time on its immobilization capacity; 10 mL MB, 40 ppm, room temperature, 0.1 g SPGMA, rpm 200, and 6.5 pH.

Variation of the adsorption time from 5 to 30 min slightly affects the adsorption capacity from 3.65 to 3.94 mg/g (Figure 6). This behavior agreed with previously published data by Mohy-Eldin et al. using amidoximated polyacrylonitrile particles [31] and OPA-pyrazole-g-PGMA particles [28] for the removal of MB dye. In addition, a very fast equilibrium was achieved due to many available exchange sites relative to the MB molecules in the liquid phase.

Three linear kinetic models were used to describe the kinetics of the sorption process and were selected in this study for describing the MB sorption process using SPGMA particles.

The pseudo-first-order kinetic model given by Langergren and Svenska [84]:

ln (q_e_ − q_t_) = ln q_e_ − k_1_t (8)

The pseudo-second-order rate (chemisorptions) is expressed as [85]:

t/q_t_ = (1/k_2_q_e_^2^) + t/q_e_ (9)

**Figure 7:**
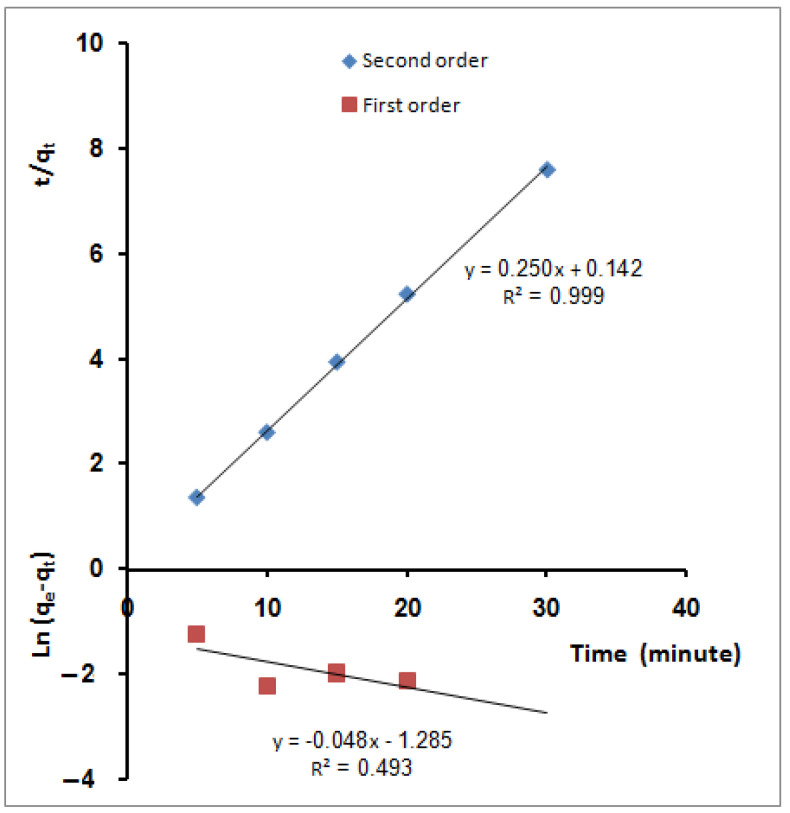
Sorption kinetic models for MB molecules’ adsorption using SPGMA; pseudo-first order and pseudo-second order.

The simple Elovich model is represented in the simple form [86]:

q_t_ = α + β ln t (10)

q_e_ and q_t_ are the amounts of ions adsorbed (mg/g) at equilibrium and time t (min), respectively. K_1_ (min^−1^) is the first-order reaction rate constant. K_2_ is the second-order reaction rate equilibrium constant (g/mg min). α represents the initial sorption rate (mg/g min) and β is related to the extent of surface coverage and activation energy for chemisorption (g/mg). The values of the first-order rate constant k_1_ and regression coefficient, R^2^, obtained from the slope of the plot ln (q_e_ − q_t_) versus time (Figure 7), are reported in Table 2. From the table, it was indicated that the correlation coefficients are not high (R^2^ = 0.493). Moreover, the estimated value of q_e_ calculated from the equation, 0.277 (mg/g), is clearly lower than the experimental value (3.94 mg/g). The pseudo-second-order kinetics applies to the experimental data in Figure 7. From the figure, the values of q_e_, calculated (4.0 mg/g), and k_2_ (0.142 g mg^−1^min^−1^) have been determined from the slope and intercept of the plot, respectively. Furthermore, the value of the regression coefficient (R^2^ = 0.999) was tabulated in Table 2. Based on linear regression values from this table (R^2^ ≈ 1), the kinetics of MB molecules’ sorption onto SPGMA nanoparticles can be aptly described by the second-order equation. Additionally, the values of q_e_ calculated resulting from the intersection points of the second-degree reaction kinetic curves (4.0 mg/g) are closer to the experimental data (3.94 mg/g) than the counterpart obtained by the pseudo-first-order model at 0.277 (mg/g). Under the studied conditions, the second-order rate expression fits the data most satisfactorily. Hence, it suggests that the rate-limiting step in these sorption processes may be chemisorptions involving influential forces through the sharing or exchanging of electrons between the sorbent and the sorbate [87].

**Figure 8:**
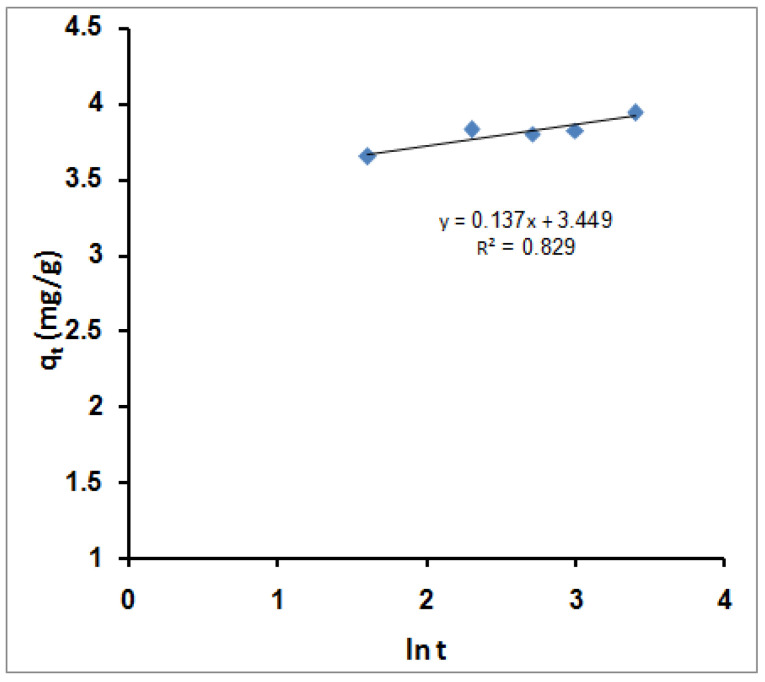
Simple Elovich sorption kinetic models for MB molecules’ adsorption using SPGMA.

Figure 8 shows the estimated Elovich equation parameters in Table 2. The value of β indicates the number of sites available for removal. At the same time, α is the removal quantity when ln t is equal to zero, i.e., the removal quantity when t is one hour (equilibrium time). This value provides insight into the removal behavior of the first step [88]. However, according to the Elovich equation, the obtained data agree with the experimental data, after the pseudo-second-order model and better than the pseudo-first-order model.

### 2.3. MB Immobilization Temperature and Adsorption Thermodynamics

**Figure 9:**
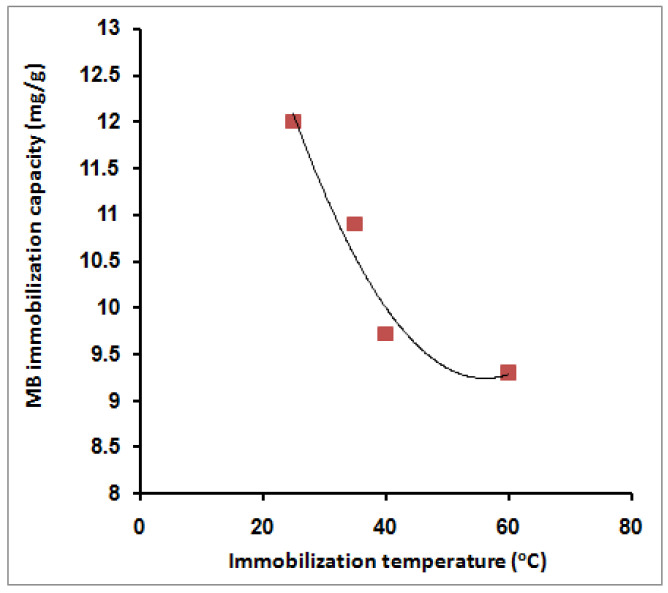
Effect of MB immobilization temperature on its immobilization capacity; 10 mL MB, 40 ppm, 0.02 g SPGMA, 5 min, rpm 200, and 6.5 pH.

Figure 9 shows the effect of varying the MB immobilization temperature on the adsorption capacity. From the figure, it is clear that elevation of the immobilization temperature has a negative effect on the MB adsorption capacity. The MB adsorption capacity was reduced from 12 mg/g to 9.3 mg/g. The negative behavior upon elevation of the temperature indicates the exothermic nature of the MB adsorption process (Figure 9). This trend agrees with theresults obtained earlier using amidoximated crosslinked polyacrylonitrile particles [31]. The obtained results are an advantage since the dye immobilization process does not need additional heating or other costs. This behavior may be referred to as the acceleration effect of temperature on the dye molecules’ adsorption on the surface of both SPGMA particles. This fast initial step reduces the concentration gradient between the MB dye liquid and polymer solid phases. ThMB concentration limitation from one side and high concentration of exchange sites over the surface of the particles on the other side contribute significantly to obtaining this behavior. The absence of a pore diffusion process also eliminates the effect of temperature [30].

The values of thermodynamic parameters should be considered to conclude the adsorption process’s spontaneity. With increasing temperature, an automatic system will display a decrease in ΔG° and ΔH° values. All the thermodynamic parameters are calculated from the following equations [89,90]:

(11) $$lnK_{d}=\frac{\Delta S}{R}-\frac{\Delta H}{RT}$$

where:

(12) $$K_{d}=\frac{q_{e}}{C_{e}}$$

(13) $$\Delta G=\;-RTlnK_{d}$$

(14) ΔH= −R×Slope

(15) ΔS=R×Intercept

**Figure 10:**
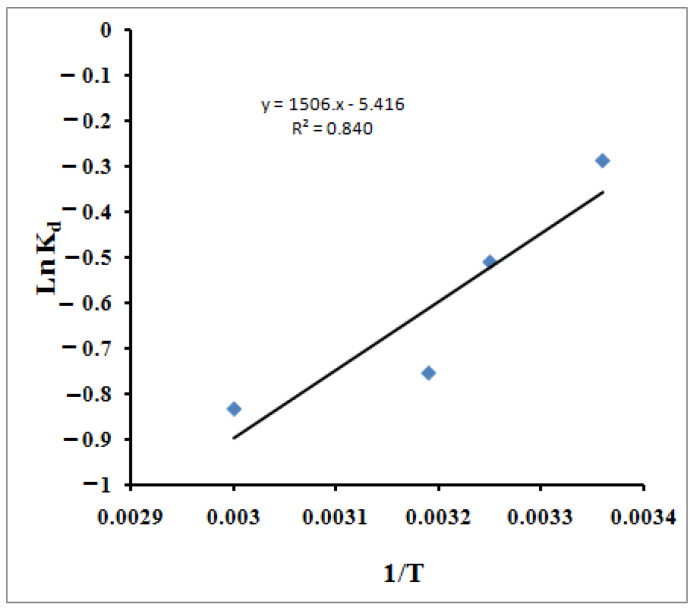
The Van’t Hoff plot of the adsorption of MB using SPGMA polymer.

*R* is the gas constant (8.314 J/mol K) and *T* is the temperature in *K*. Table 3 lists the values for the thermodynamic parameters (Figure 10). The negative value for the Δ*H*° (−12.52 kJ/mol) indicates the exothermic nature of the process, which explains the decrease in the MB molecules’ adsorption capacity as the temperature increased. The enthalpy change in the chemisorption process (40–120 kJ mol^−1^) is more significant than the physisorption change [91]. Consequently, the obtained value of the heat of adsorption acquired in this study, −12.52 kJ mol^−1^, indicates that the adsorption of the MB cations is likelyattributable to the physisorption in accordance with the kinetics study, which described the adsorption as a mix between chemisorption and physisorption. Thus, it is evident from the lower Δ*H*° value that the physisorption also takes part in the adsorption process. The MB molecules adhere to the adsorbent surface only through weak intermolecular interactions. The Δ*G*° values reflect the feasibility of the process. The negative value for the entropy change, Δ*S*° (−45.03 J/mol K), illustrates the increment of the orderliness at the solid/liquid interface resulting from the adsorption of the MB molecules. Kifuani et al. [92] studied the preparation of a bioadsorbent from the seeds of *Cucumeropsis mannii* Naudin (BCM) and examined its effectiveness in the removal of methylene blue (MB) from aqueous solution by adsorption process. Thermodynamics parameters of the adsorption process were determined. They found that the negative values of Δ*H*°, higher than 41 kJ mol^−1^, show that the adsorption of MB on BCM is exothermic and essentially a chemical process. A negative value of entropy, Δ*S*°, indicates that the disorder of the molecules decreases in the interface between the MB dye and BCM bioadsorbent. Moreover, the standard free enthalpy hasa positive value, which indicates a non-spontaneous adsorption process. Wu et al.’spublished resultsconcerned the adsorption of MB onto the bioadsorbent spent substrate of *Pleurotus eryngii* (SSPE) [93]. They found that Δ*H°* and Δ*S*° were all negative. The obtained value of Δ*S*° (−42.6 J/mol K) is very close to the result obtained in our study. They claimed the obtained negative enthalpy was a result of the exothermic process of the dye’s adsorption onto SSPE, while the negative entropy was attributed to the decreased degree of system chaos because the dissolved dyes were adsorbed onto SSPE. The Δ*G*° of methylene blue adsorbed onto SSPE were all negative, which indicated that the adsorption process was a spontaneous process. Yagub et al. [94] studied the adsorption capacity of raw and sodium hydroxide-treated pinecone powder in the removal of methylene blue (MB) from aqueous solution The temperature-dependent performance of MB adsorption was further analyzed based on the thermodynamic parameters, such as the change in free energy Δ*G°*, enthalpy Δ*H*°, and entropy Δ*S*°. Δ*G*° ranged from −13.64 to −12.25 kJ mole^−1^, depending on the temperature, which ranged from 303 to 323 K. It was observed that the Δ*G*° values at all temperatures were negative. It indicates that the MB dye adsorption reaction is spontaneous with the temperatures studied. The value of Δ*G*° increases, thus indicating that the adsorption of MB on the pinecone became more favorable at lower temperatures. The negative value of Δ*H*° indicates that the sorption process was exothermic, whereas the negative value of Δ*S*° indicated decreased randomness at the solid–solute interface as a result of the MB adsorption.

The interpretation of the contradictory findings of thermodynamic (physisorption, enthalpy) and kinetics (chemisorption, rate laws) can be declared according to the fact that the kinetic laws were applied ata fixed temperature, where the chemisorption takes place in the SPGMA region with a higher rate than the physisorption (enthalpy) that takes place in the PGMA region, which is larger. When using higher temperatures, the part of MB molecules that is chemisorbed is limited by the fixed number of negative sulfonic adsorption sites, which were already covered at the lowest used temperature (25 °C).A further increase in the immobilization (adsorption) temperature has a negative linear effect up to 40 °C, while the effect was reduced with a further increment of the temperature up to 60 °C due to the exothermic nature of the chemisorption process. The obtained results indicate that the chemisorption contributes approximately 25% in the MB adsorption process. On the other hand, the MB molecules immobilized (adsorbed) by physisorption, which is the larger part of immobilized MB, were not affected by the increase in temperature. Such an explanation is supported by the obtained adsorption capacity behavior shown in Figure 9.

### 2.4. Simulation Mathematical Model

**Figure 11:**
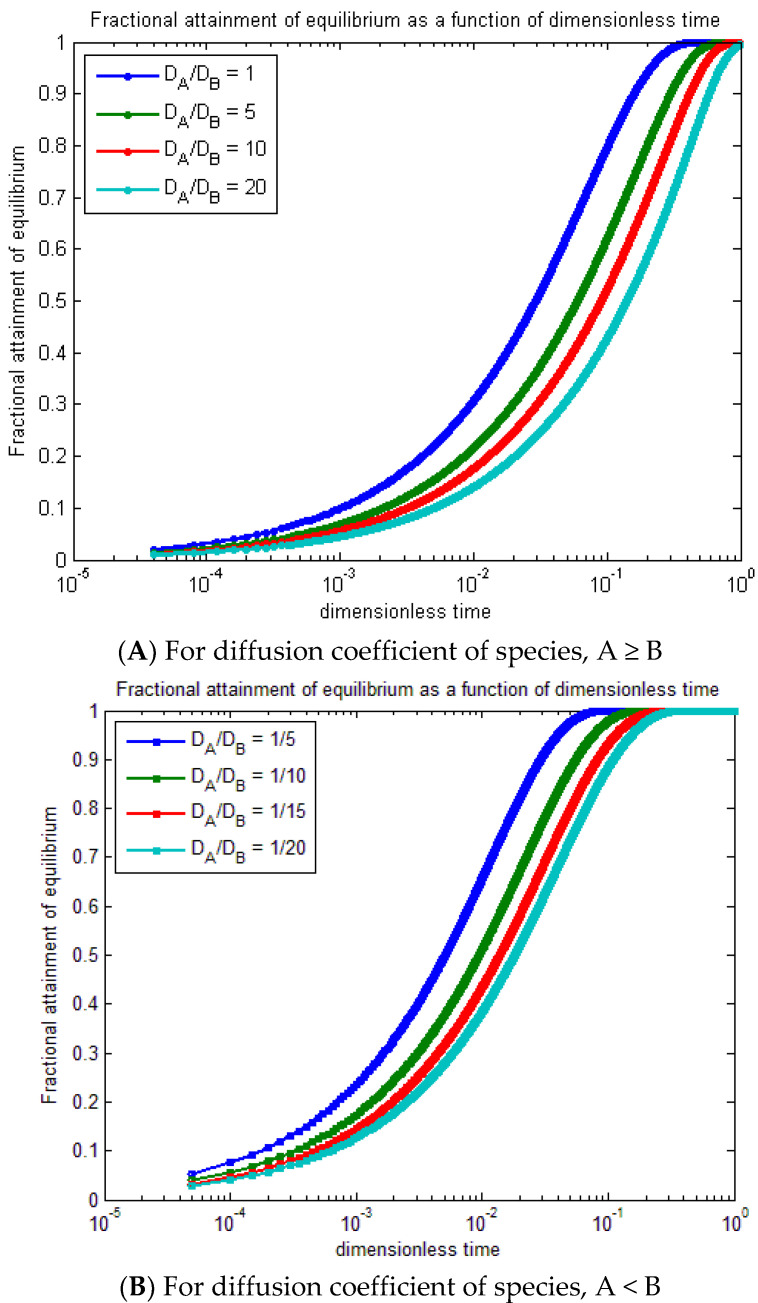
Equilibrium fractional attainment as a function of dimensionless time for $\frac{Z_{A}}{Z_{B}}=1$ for different diffusion coefficients (**A**,**B**).

The radial concentration profiles of MB (species A) into polymer particles (species B) were demonstrated by a dimensionless function, and the fractional attainment of equilibrium was estimated with the mentioned initial and boundary conditions that depend upon the polymer particle size, diffusion constant of MB, and MB concentration. Figure 11 shows the variation of equilibrium fractional attainment versus dimensionless time that depends on the diffusion constant of MB; therefore, it was used for a comparison between the ion exchange processes.

Figure 11A represents the fractional attainment of equilibrium versus time for the diffusion coefficient of species A ≥ B, and D_A_/D_B_ ≥ 1 is used to compare the ion exchange. As time increases, the fractional attainment of equilibrium increases by decreasing the D_A_/D_B_ as the fractional attainment of equilibrium increases. In the range of 10^−3^ to 10^−4^, the trend slightly increases from 10^0^ to 10^−3^ as the curves rapidly increase. Figure 11B shows the fractional attainment of equilibrium versus time for the diffusion coefficient of species, A < B, and DA/DB 1 from $\frac{1}{5}\;\mathrm{to}\;\frac{1}{20}$. By increasing the diffusion coefficient of spicy B, the fractional attainment of equilibrium decreases at $\frac{Z_{A}}{Z_{B}}=1$.

**Figure 12:**
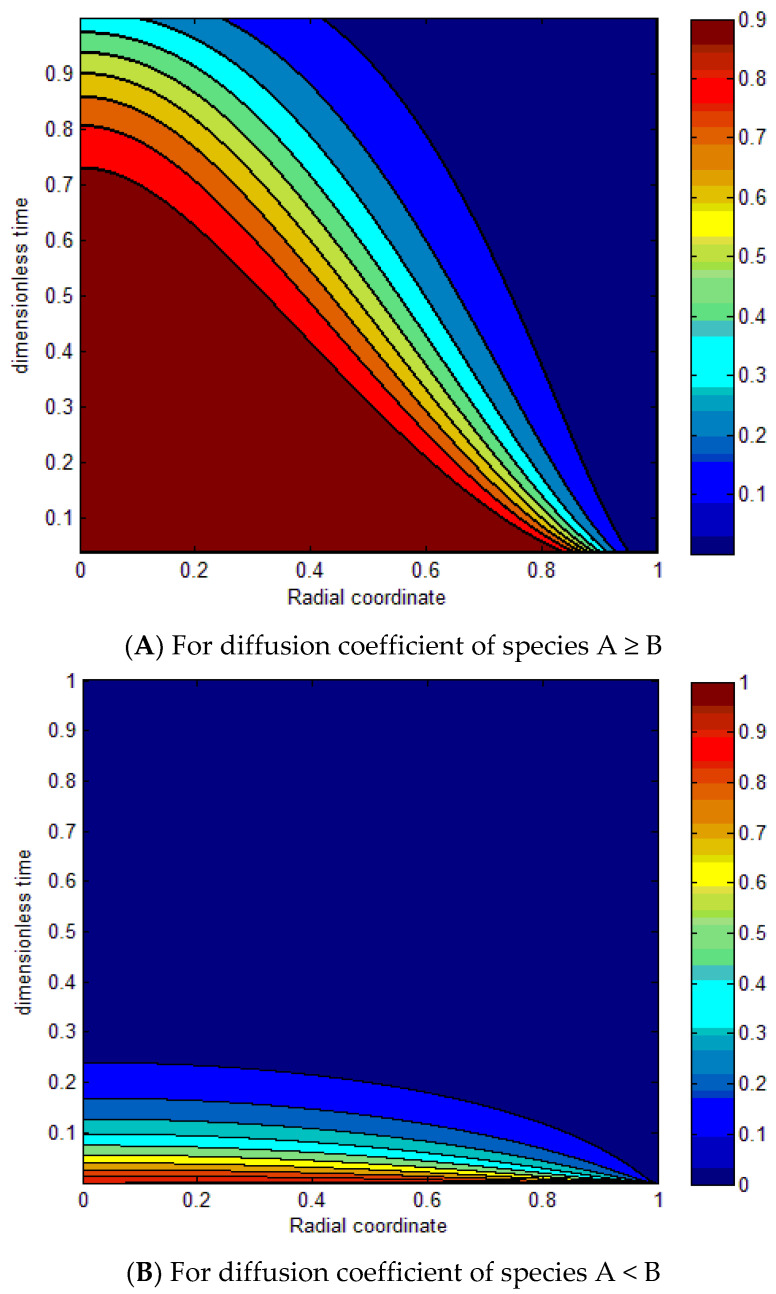
Dimensionless function α(X.τ) for $\frac{Z_{A}}{Z_{B}}=1$ for different diffusion coefficients (**A**,**B**).

Figure 12A demonstrates the contours of the radial concentration profiles of species A as a function of the radial coordinate and dimensionless time for both processes with different D_A_/D_B_ ratios and different valences. The results indicate that the curves increase as D_A_/D_B_ increases, of species A ≥ B with $\frac{Z_{A}}{Z_{B}}=1$. Figure 12B determines the contours of the radial concentration profiles of species B as a function of the radial coordinate and dimensionless time for both processes with different D_A_/D_B_ ratios and different valences. The results specify that the curves decrease by decreasing the D_A_/D_B_ of species A < B with $\frac{Z_{A}}{Z_{B}}=1$.

**Figure 13:**
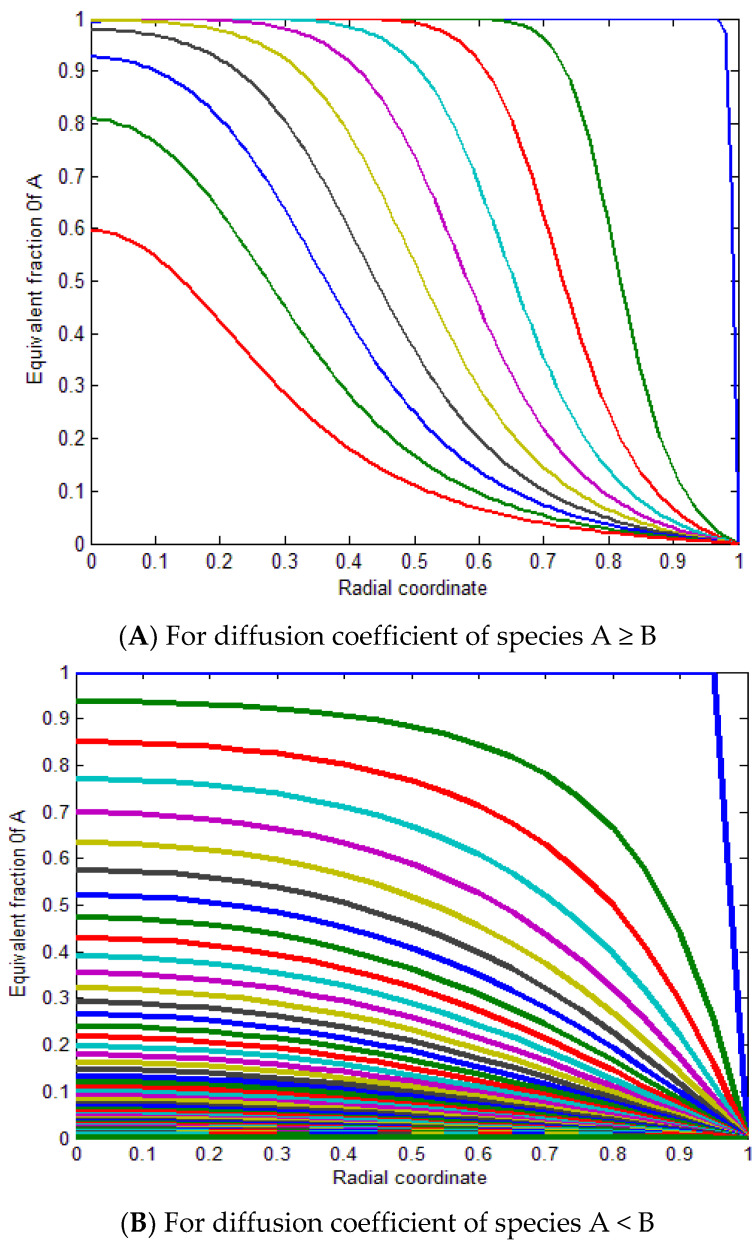
Equivalent fraction of A versus radial coordinate for $\frac{Z_{A}}{Z_{B}}=1$ for different diffusion coefficients (**A**,**B**).

Figure 13A represents the equivalent fraction of A versus the radial coordinate for ZA/ZB = 1 for diffusion coefficients of species A ≥ B and (B) for diffusion coefficients of species A < B. In conclusion, the mathematical simulation model indicates that the ion exchange process performance between the MB ions and the ion exchange sites over the SPGMA matrix decreased according to the decline in the fractional attainment of the equilibrium with a change in the diffusion coefficient ratio from 1:1. Therefore, the ideal case is when the diffusion coefficient percentage is close to one.

### 2.5. Metal Ions Removal from Wastewater

The selected MB-SPGMA composite adsorbent with a composition of 27.32 mg/g has been used for the first time in treating synthetic-contaminated water with dichromate (Cr^6+^) or permanganate (Mn^7+^) ions under batch conditions. Synthetic-contaminated water with various metal ions concentrations, 2–8 ppm, was used in the study. The removal percentage of both ions is illustrated in Table 4.

The table shows that the affinity of the MB-SPGMA composite adsorbent increased to remove the metal ions from the contaminated water increases with an increase in the metal ions’ concentration. The removal percentage in the case of permanganate ions is higher than the dichromate counters ions, especially at higher metal ion concentrations.

## 3. Materials and Methods

### 3.1. Materials

Glycidyl methacrylate (GMA) waspurchased from ACROS (USA). Potassium persulfate (KPS) and sodium disulphite (SDS) were obtained from Sigma Chem. Co. (St. Louis, MO, USA). Ethanol absolute was purchasedfrom Adwic, Egypt, and finally, MB was purchased from Aldrich, Germany.

### 3.2. Polymerization Process

Glycidyl methacrylate was polymerized under fixed conditions to prepare poly(glycidyl methacrylate) [64]. First, the 10% (*v*/*v*) monomer was dissolved in 0.05 M KPS solution in ethanol/water (1:1) and mixed well. Next, the mixture was kept in awater bath at 60 °C for 3 h to polymerize and then left overnight at room temperature while the precipitate of poly(glycidyl methacrylate) (PGMA) was formed. Next, the formed polymer was filtered and successively washed with an ethanol/water solution to remove the unreacted monomer and initiator. Finally, the polymer was dried overnight at 80 °C. The polymerization process was highly efficient, and the yield reached almost 100%.

### 3.3. Sulphonation Process

The PGMA was functionalized with negative sulphonic groups as follows. First, 0.5 g of the polymer was reacted with 20 mL of a 3% sodium sulfite (S.S.) solution (ethanol/water) at room temperature for one hour. The sulphonated polymer (SPGMA) was then washed with ethanol/water to remove unreacted S.S. The polymer was then dried at 60 °C overnight. The SPGMA was characterized using FTIR, TGA, and SEM [29]. The average particle size of SPGMA was 430 nm [66].

### 3.4. Preparation of Basic Methylen Blue Solution

A methylene blue (MB) stock solution was prepared by dissolving 0.1 g in 1000 mL of distilled water using a magnetic stirrer. The MB concentration in the supernatant and residual solutions was determined by measuring their absorbance in a 1 cm light-path cell at a maximum wavelength of 665 nm using a UV-visible spectrophotometer (T70+ PG Instruments).

### 3.5. Standard Curve of MB Concentration

**Figure 14:**
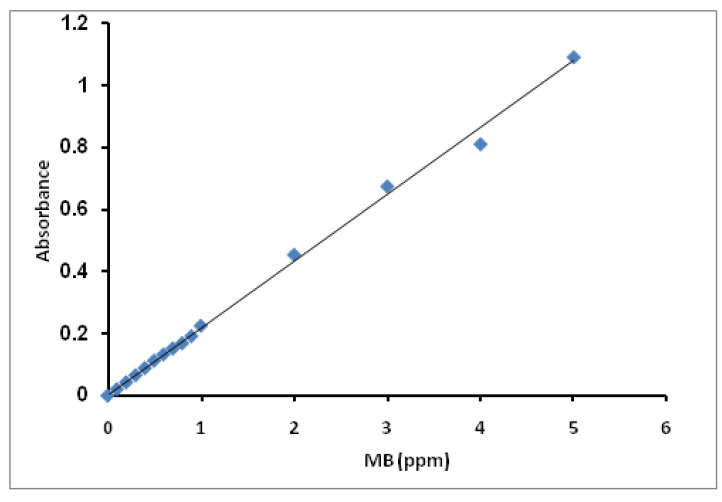
Standard curve for MB concentration.

Varied MB solution concentrations from 0.1 ppm to 5 ppm were prepared. The samples’ absorbance (A_abs_) was measured using a UV-Visible spectrophotometer and plotted against their concentrations. From the slope, we can derivative the constant, equal to (1/slope). The standard curve of MB concentrations is presented in Figure 14. The constant has been calculated from the curve’s slope and was found to be 4.65.

### 3.6. Methylene Blue–Polymers Composite Formation (Immobilization Process)

The methylene blue–polymers composite formation process was performed through immobilization experiments in a batch process using an MB aqueous solution.

To study the MB concentration effect, the MB immobilization was performed by mixing 0.1 g of SPGMA-based polymers with 10 mL of 10–40 ppm MB of pH 6.5. The mixture was agitated using a magnetic stirrer at 200 rpm at room temperature for 30 min and then centrifuged at 12,000 rpm for 30 min to separate the matrix of the liquid phase.

To study the MB immobilization time effect, the MB immobilization was performed by mixing 0.1 g of SPGMA-based polymers with 10 mL of 40 ppm MB of pH 6.5. The mixture was agitated using a magnetic stirrer at 200 rpm at room temperature for 5–30min and then centrifuged at 12,000 rpm for 30 min to separate the matrix of the liquid phase.

To study the effect of the MB immobilization temperature, the MB immobilization was performed by mixing 0.02 g of SPGMA-based polymers with 10 mL of 40 ppm MB of pH 6.5. The mixture was agitated using a magnetic stirrer at 200 rpm at temperatures ranging from 25 to 60 °C for 5 min and then centrifuged at 12,000 rpm for 30 min to separate the matrix of the liquid phase.

The remaining MB concentration (C_t_ and/or C_e_; ppm) in the liquid phase after the immobilization process was determined by measuring the absorbance at the maximum wavelength (ʎ_max_ = 665 nm) using a UV-VIS spectrophotometer (T70+ PG Instruments) and multiplied by a 4.65 constant extracted from the slope of the standard curve.

The MB–polymers composites composition (mg/g) was calculated according to the following formula:

MB immobilization capacity (q_e_ and/or q_max_; mg/g) = V (C_0_ − C_t_)/M (16)

where C_0_ and C_t_ are the MB initial and final concentrations at definite immobilization times, V is the volume of the MB solution (L), and M is the mass of the SPGMA polymers (g).

### 3.7. Isotherm, Kinetic, and Thermodynamic Studies

The MB immobilization process via adsorption onto SPGMA particles has been characterized using isotherm models, namely, Freundlich, Langmuir, D–R [73,74,75,76], and Temkin isotherm models [82,83]. The data used in the calculation of the different isotherm parameters and constants are summarized in Table 5.

The kinetics of the MB adsorption process followed three linear kinetic models, namely, pseudo-first-order, pseudo-second-order, and Elovich models [84,85,86]. The data used in the calculation of the kinetic models’ parameters are summarized in Table 6.

Finally, the thermodynamic parameters, Δ*G*, Δ*H*, and Δ*S*, of the MB adsorption process were investigated using the Van’t Hoff model [89,90,91]. The data used in the calculation of the thermodynamic parameter, Δ*G*, are summarized in Table 7.

### 3.8. Mathematical Model of the MB Immobilization Process *[95]*

We consider the ion exchange between ion exchanger SPGMA of a postulated uniform size (430 nm) containing the counter ions’ exchange sites (A) and the well-stirred solution containing the MB counter species (B). During the immobilization (adsorption) process, species (B) diffused into species (A).

The governing equation that describes the ion exchange resin is derived from Nernst–Plank equations between two ion species [A] and [B]. The ion species fluxes of [A] and [B] are given in Equations (17) and (18). A spherical coordinate system is used in 1-D under spherical symmetry and then the dimensionless form of Equation (19) is derived from (17) and (18). The equations written by applying these assumptions to the porous structure of the resin are neglected and the resin will be treated as a quasi-homogeneous phase. The concentration of the ion groups is assumed to be constant.

(17) Ψ_A=−D_A [gradC_A+Z_AC_A (F/RT)gradφ]

(18) $$\Psi_{B}=-D_{B}\left[gradC_{B}+Z_{B}C_{B}\left(\frac{F}{RT}\right)grad\varphi \right]$$

(19) $$\frac{\partial \gamma }{\partial \tau }-\frac{1}{\rho^{2}}\frac{\partial }{\partial \rho }\left[\frac{1+b\gamma }{1+a\gamma }\rho^{2\;}\frac{\partial \gamma }{\partial \rho }\right]=0$$

(20) $$\gamma =\frac{Z_{A}C_{A}}{C}\tau =\frac{D_{A}t}{r_{o}^{2}}\rho =\frac{r}{r_{o}}$$

where [Ψ] is the flux for [A] and [B], [D] is the individual diffusion constant for [A] and [B], [R] is the gas constant, [T] is the absolute temperature, [F] is the Faraday constant, [φ] is the electric potential, $\left[C_{i}\right]$ is the molar concentration of species i, $\left[Z_{i}\right]$ is the valence of species i, [r] is the radial spherical coordinate, and $\left[r_{o}\right]$ is the particle radius. The finite difference method is used to discretize Equation (19) as follows:

(21) $$\left(\rho .\tau +\Delta \tau \right)=\gamma \left(\rho .\tau \right)+\frac{\Delta \tau }{\Delta \rho^{2}}\left\{R_{+}D_{+}\left[\gamma \left(\rho +\Delta \rho .\tau \right)-\gamma \left(\rho .\tau \right)\right]-R_{-}D_{-}\left[\gamma \left(\rho .\tau \right)-\gamma \left(\rho -\Delta \rho .\tau \right)\right]\right\}$$

(22) $$R_{+}\left(\rho \right)={\left[\frac{\rho +\frac{1}{2}\Delta \rho }{\rho }\right]}^{2}$$

(23) $$R_{-}\left(\rho \right)={\left[\frac{\rho -\frac{1}{2}\Delta \rho }{\rho }\right]}^{2}$$

(24) $$D_{+}\left(\gamma \right)=\frac{2+b\left[\gamma \left(\rho +\Delta \rho .\tau \right)+\gamma \left(\rho .\tau \right)\right]}{2+a\left[\gamma \left(\rho +\Delta \rho .\tau \right)+\gamma \left(\rho .\tau \right)\right]}$$

(25) $$D_{-}\left(\gamma \right)=\frac{2+b\left[\gamma \left(\rho .\tau \right)+\gamma \left(\rho -\Delta \rho .\tau \right)\right]}{2+a\left[\gamma \left(\rho .\tau \right)+\gamma \left(\rho -\Delta \rho .\tau \right)\right]}$$

where [a] and [b] are constants

(26) $$a=\frac{Z_{A}D_{A}}{Z_{B}D_{B}}-1\;\;\;\;\;\;\;\;\;\;\;\;\;\;and\;b=\frac{Z_{A}}{Z_{B}}-1\;\;\;\;\;$$

(27) $$-\frac{dQ_{A}}{dt}=-\frac{VCD_{A}}{r_{o}{}^{2}}\frac{dq_{A}}{d\tau }$$

(28) $$q_{A}\left(\tau \right)=3\overset{1}{\underset{0}{\int }}\gamma \left(\rho .\tau \right)\rho^{2\;}d\rho$$

where $\left[q_{A}\right]\;$ is the amount of species A in the bead and [V] is the bead volume. According to Simpson’s rule, the equations are represented as follows:

(29) $$q_{A}\left(\tau \right)={\left(\Delta \rho \right)}^{3}\left[4\overset{\frac{1}{\Delta \rho }-1}{\underset{n=1.3.5}{\sum }}n^{2}\gamma \left(\rho .\tau \right)+2\overset{\frac{1}{\Delta \rho }-2}{\underset{n=2.4.6}{\sum }}n^{2}\gamma \left(\rho .\tau \right)\right]$$

where

(30) $$\;n=\frac{\rho }{\Delta \rho }$$

(31) $$F\left(\tau \right)=1-q_{A}\left(\tau \right)$$

where F(τ) represents the equilibrium fractional attainment. To solve Equation (18), the initial and boundary conditions are applied as follows

(32) γ(ρ.τ=0)=1                        0≤ρ<1  

(33) γ(ρ=1.τ)=0           and γ(ρ=0.τ)=γ(ρ=Δρ.τ)

The stability condition for the discretized equation is

(34) $$\Delta \tau =0.4\;\frac{\Delta \rho^{2}}{D_{max}}$$

(35) $$D_{max}=1\;\;\;\;\;\;\;\;\;\;\;\;\;\;\;\;\;\;\;\;if\;a>0\;\;\;.b<0\;\;\;\;\;\;\;\;\;\;\;\;\;\;\;\;\;\;\;$$

(36) $$D_{max}=\frac{1+b}{1+a}if\;a<0\;\;\;.b>0\;\;\;\;\;\;\;\;\;\;\;$$

### 3.9. Chromium (VI) and Manganese (VII) Ion Removal *[70,71]*

Synthetic dichromate, Cr^6+^, and permanganate, Mn^7+^, 20 mL solutions with varying concentrations (2–8 ppm) were mixed with 0.1 g of the MB-SPGMA composite at room temperature for 3 h and were then separated by centrifugation under 12,000 rpm for 30 min in a batch adsorption experiment. The Cr^6+^ and Mn^7+^ concentrations (ppm) before and after the adsorption for each solution were determined by measuring the absorbance at a maximum wavelength (ʎ_max_ = 380 nm and 550 nm) using a UV-VIS spectrophotometer (T70+ PG Instruments) and multiplying it by the constant extracted from the slope of the standard curve. The adsorption capacity was calculated according to the following equation:

Metal ions removal percentage (%) = [(C_M0_ − C_Mt_)/C_M0_] × 100 (37)

C_M0_ and C_Mt_ are the metal ions’ initial and final concentrations at a defined adsorption time.

## 4. Conclusions

The methylene blue–sulphonated poly(glycidyl methacrylate) polymer composite novel adsorbent was developed through an adsorption technique. The MB content of the MB-polymer composite was monitored with the variation of the MB concentration, MB immobilization time, and temperature. The MB immobilization capacity has a direct linear relationship with the MB concentration. The MB immobilization process via adsorption onto SPGMA particles has been characterized using other models, namely, Freundlich, Langmuir, D–R, and Temkin isotherm models. The data best fit the Freundlich isotherm model, which postulates heterogeneous surface site energies and multi-layer levels of sorption. The immobilization process was speedy, and more than 90% of MB was immobilized within 5 min. The kinetics of the MB adsorption process followed three linear kinetic models: Pseudo-first-order, pseudo-second-order, and Elovich models. The data were found to follow the pseudo-second-order model followed by the pseudo-first-order model assuming the coexistence of both chemisorption and physisorption of the MB onto SPGMA and PGMA, respectively. The elevation of the immobilization temperature from 25 to 60 °C reduced the MB content of the MB-SPGMA composites by roughly 25%. Finally, the MB adsorption process’ thermodynamic parameters, Δ*G*, Δ*H*, and Δ*S*, were investigated using theVan’t Hoff model. The negative value for the Δ*H*° (−12.52 kJ/mol) indicates the exothermic nature of the process, which explains the decrease inthe MB molecules’ adsorption capacity as the temperature increased. The enthalpy change inthe chemisorption process (40–120 kJ mol^−1^) is more significant than the physisorption. Consequently, the obtained value of the heat of adsorption acquired in this study, −12.52 kJ mol^−1^, indicates that the adsorption of the MB cations is like lyattributable to physisorption, which is not in accordance with the kinetics study, which described that the adsorption is chemisorption. Thus, it is evident from the lower Δ*H*° value that the physisorption also takes part in the adsorption process, mainly in the PGMA region. The MB molecules adhere to the adsorbent surface only through weak intermolecular interactions. The negative value for the entropy change, Δ*S*° (−45.03 J/mol K), illustrates the orderliness at the solid/liquid interface as a result of the adsorption of the MB molecules. The Δ*G*° values reflect the feasibility of the process. Finally, the mathematical simulation model indicates that the ion exchange process performance between the MB ions and the ion exchange sites over the SPGMA matrix decreased according to the decline in the fractional attainment of the equilibrium with a change in the diffusion coefficient ratio from 1:1. Therefore, the ideal case is when the diffusion coefficient percentage is close to one. The selected MB-SPGMA composite adsorbent with a composition of 27.32 mg/g was used for the first time to treat synthetic-contaminated water with dichromate or permanganate under batch conditions. The obtained data declared that the affinity of the MB-SPGMA composite adsorbent increased to remove the metal ions from the contaminated water with the increase in the metal ion concentration. On the other hand, the removal percentage in the case of Mn^7+^ ions is higher than the Cr^6+^ counter ions, especially at higher metal ion concentrations.

## Figures and Tables

**Table 1:** R_L_ values for different initial MB molecules concentrations.

C_o_	R_L_
5	0.137
10	0.0737
15	0.0504
20	0.0382
30	0.0258

**Table 2:** The adsorption parameters of the pseudo-first- and pseudo-second-order and the Elovich kinetic models.

Adsorbent	Pseudo-First-Order				Pseudo-Second-Order			Elovich		
	q_e,exp,_ (mg/g)	q_e,cal,_ (mg/g)	K_1_ (min^−1^)	R^2^	q_e,cal_ (mg/g)	K_2_ (g mg^−1^min^−1^)	R^2^	β (g/mg)	α (mg/g min)	R^2^
SPGMA	3.94	0.277	0.048	0.493	4.00	0.1422	0.999	0.137	3.449	0.829

**Table 3:** Thermodynamic parameter valuesof the MB molecules adsorption on the SPGMA under different temperatures.

1/T	Δ*G* (kJ/mol)	Δ*H* (kJ/mol)	Δ*S* (J·mol^−1^·K^−1^)
0.00336	0.713	−12.52	−45.03
0.00325	1.309		
0.00319	1.965		
0.00300	2.309		

**Table 4:** Removal percentage of dichromate (Cr^6+^) and/or permanganate (Mn^7+^) ions from contaminated wastewater under batch conditions.

Metal Ions Concentration (ppm)	Metal Ions Removal Percentage (%)	
	Cr^6+^	Mn^7+^
**2**	16.5	11
**4**	20	22
**6**	25	38
**8**	36	55

**Table 5:** The q_e_ (mg/g) and C_e_ (mg/L) values for different MB concentrations.

MB (ppm)	q_e_ (mg/g)	C_e_ (mg/L)
**8**	0.761	0.392
**16**	1.545	0.5536
**24**	2.328	0.72
**32**	3.118	0.80
**40**	3.916	0.88

**Table 6:** The values used in the calculation of kinetic models’ parameters and constants.

Time (t)	lnt	Capacity (q_t_)	t/q_t_	ln(q_e_ − q_t_)
**5**	1.61	3.65	1.37	−1.24
**10**	2.3	3.83	2.61	−2.21
**15**	2.71	3.8	3.95	−1.97
**20**	3	3.82	5.24	−2.12
**30**	3.4	3.94	7.61	

**Table 7:** The values used in the calculation of thermodynamic parameters and constants.

Temperature (°C)/(K)	q_e_	C_e_	K_d_	ln K_d_
**25/298**	12	16	0.75	−0.288
**35/308**	10.9	18.2	0.6	−0.511
**40/313**	9.71	20.576	0.47	−0.755
**60/333**	9.3	21.412	0.43	−0.834

## Data Availability

Not applicable.

## Sample Availability

Samples of the compounds are not available from the authors.

## References

[1] Gómez V., Larrechi M., Callao M. (2007). Kinetic and adsorption study of acid dye removal using activated carbon. Chemosphere.

[2] Gupta V.K., Kumar R., Nayak A., Saleh T.A., Barakat M.A. (2013). Adsorptive removal of dyes from aqueous solution onto carbon nanotubes: A review. Adv. Colloid Interface Sci..

[3] Ayad M.M., Abo El-Nasr A. (2010). Adsorption of cationic dye (methylene blue) from water using polyaniline nano-tubes base. J. Phys. Chem. C.

[4] Wong Y.C., Szeto Y.S., Cheung W.H., McKay G. (2003). Equilibrium Studies for Acid Dye Adsorption onto Chitosan. Langmuir.

[5] Baybars A.F., Cengiz Q., Mustafa K. (2012). Cationic dye (methylene blue) removal from aqueous solution by montmorillonite. Bull. Korean Chem. Soc..

[6] Yagub M.T., Sen T.K., Ang H.M. (2012). Equilibrium, Kinetics, and Thermodynamics of Methylene Blue Adsorption by Pine Tree Leaves. Water Air Soil Pollut..

[7] Sen T.K., Afroze S., Ang H.M. (2011). Equilibrium, kinetics and mechanism of removal of methylene blue from aqueous solution by adsorption onto pine cone biomass of Pinus radiate. Water Air Soil Pollut..

[8] Mohammad M., Maitra S., Ahmad N., Bustam A., Sen T., Dutta B.K. (2010). Metal ion removal from aqueous solution using physic seed hull. J. Hazard. Mater..

[9] Abd EI-Latif M.M., Ibrahim A.M., EI-Kady M.F. (2010). Adsorption equilibrium, kinetics and thermodynamics of methylene blue from aqueous solutions using biopolymer oak sawdust composite. J. Am. Sci..

[10] Yao Z., Wang L., Qi J. (2009). Biosorption of Methylene Blue from Aqueous Solution Using a Bioenergy Forest Waste: *Xanthoceras sorbifolia*Seed Coat. Clean. Soil Air Water.

[11] Yagub M.T., Sen T.K., Afroze S., Ang H.M. (2014). Dye and its removal from aqueous solution by adsorption: A review. Adv. Colloid Interface Sci..

[12] Salleh M.A.M., Mahmoud D.K., Karim W.A.W.A., Idris A. (2011). Cationic and anionic dye adsorption by agricultural solid wastes: A comprehensive review. Desalination.

[13] Srinivasan A., Viraraghavan T. (2010). Decolorization of dye wastewaters by biosorbents: A review. J. Environ. Manag..

[14] Leszczynska M., Hubicki Z. (2009). Application of weakly and strongly basic anion exchangers for the removal of brilliant yellow from aqueous solutions. Desalin. Water Treat..

[15] Purkait M., Maiti A., DasGupta S., De S. (2007). Removal of congo red using activated carbon and its regeneration. J. Hazard. Mater..

[16] Hernández-Montoya V., Pérez-Cruz M., Mendoza-Castillo D., Moreno-Virgen M., Bonilla-Petriciolet A. (2013). Competitive adsorption of dyes and heavy metals on zeolitic structures. J. Environ. Manag..

[17] Errais E., Duplay J., Elhabiri M., Khodja M., Ocampo R., Baltenweck-Guyot R., Darragi F. (2012). Anionic RR120 dye adsorption onto raw clay: Surface properties and adsorption mechanism. Colloids Surf. A Physicochem. Eng. Asp..

[18] Ofomaja A.E. (2009). Equilibrium sorption of methylene blue using mansonia wood sawdust as biosorbent. Desalin. Water Treat..

[19] Reddy M.S., Sivaramakrishna L., Reddy A.V. (2012). The use of an agricultural waste material, Jujuba seeds for the removal of anionic dye (Congo red) from aqueous medium. J. Hazard. Mater..

[20] Sarioglu M., Atay U.A. (2006). Removal of Methylene blue by using biosolid. Global Nest J..

[21] Deniz F., Karaman S. (2011). Removal of Basic Red 46 dye from aqueous solution by pine tree leaves. Chem. Eng. J..

[22] Dawood S., Sen T.K. (2012). Removal of anionic dye Congo red from aqueous solution by raw pine and acid-treated pine cone powder as adsorbent: Equilibrium, thermodynamic, kinetics, mechanism and process design. Water Res..

[23] Auta M., Hameed B. (2013). Coalesced chitosan activated carbon composite for batch and fixed-bed adsorption of cationic and anionic dyes. Colloids Surf. B Biointerfaces.

[24] Poinern G.E.J., Senanayake G., Shah N., Thi-Le X.N., Parkinson G.M. (2011). Adsorption of the aurocyanide, View the MathML source complex on granular activated carbons derived from macadamia nut shells—A preliminary study. Miner. Eng..

[25] Garba A., Tahir A., Yusuf A.K., Al-Qalam University Katsina (2021). Adsorption of Methylene Blue Using Activated Carbon Made from Watermelon Rinds. J. Sustain. Mater. Process. Manag..

[26] Eldin M.M., Gouda M., Abu-Saied M., El-Shazly Y.M., Farag H. (2016). Development of grafted cotton fabrics ions exchanger for dye removal applications: Methylene blue model. Desalination Water Treat..

[27] Mohy Eldin M.S., Elkady M.F., Abdel Rahman A.M., Soliman E.A., Elzatahry A.A., Youssef M.E., Eweida B.Y. (2012). Preparation and characterization of imino diacetic acid functionalized alginate beads for removal of contaminates from waste water: I. methylene blue cationic dye model. Desali. Water Treat..

[28] Mohy E.M.S., Aly K., Khan Z.A., Meky A.E., Saleh T.S., Elbogamy A.S. (2016). Development of Novel Acid-Base Ions Exchanger for Basic Dye Removal: Phosphoric Acid Doped Pyrazole-g-Polyglycidyl Methacrylate. Desali. Water Treat..

[29] Elkady M.F., Mohy Eldin M.S., Abu-Saied M.A., Abdel Rahman A.M., Soliman E.A., Elzatahry A.A., Youssef M.E. (2011). Novel nano-sulphonated polyglycidyl methacrylate cation exchanger for removal of heavy metals: Optimization of the operational conditions. Desalination.

[30] Mohy Eldin M.S., El-Sakka S.A., El-Masry M.M., Abdel-Gawad I.I., Garybe S.S. (2012). Removal of methylene blue dye from aqueous medium by nano-polyacrylonitrile particles. Desalin. Water Treat..

[31] Mohy Eldin M.S., Aggour Y.A., El-Aassar M.R., Beghet G.E., Atta R.R. (2016). Development of nano-crosslinked polyacrylonitrile ions exchanger particles for dyes removal. Desalin. Water Treat..

[32] Eldin M.M., Abu-Saied M., Tamer T., Youssef M., Hashem A., Sabet M. (2016). Development of polystyrene based nanoparticles ions exchange resin for water purification applications. Desalin. Water Treat..

[33] Aksu Z., Wong Y.-S., Tam N.F.Y. (1998). Algae for wastewater treatment. Biosorption of Heavy Metals by Microalgae in Batch and Continuous Systems.

[34] Dönmez G., Aksu Z. (1999). The effect of copper(II) ions on the growth and bioaccumulation properties of some yeasts. Process Biochem..

[35] Das J., Dangar T.K., Panigrahy M. (2022). Bioremediation of Heavy Metals: A Substantive Potential for Clean Earth. J. Sustain. Mater. Process. Manag..

[36] (2008). Ahmaruzzaman Adsorption of phenolic compounds on low-cost adsorbents: A review. Adv. Colloid Interface Sci..

[37] Waldemer R.H., Tratnyek P.G. (2005). Kinetics of Contaminant Degradation by Permanganate. Environ. Sci. Technol..

[38] Gupta V., Kumari S., Virvadiya C. (2015). Adsorption Analysis of Mn(VII) from Aqueous Medium by Activated Orange Peels Powder. Int. Res. J. Pure Appl. Chem..

[39] Zhang K., Li C., He J., Liu R. (1997). Adsorption of permanganate onto activated carbon particles. Hua Xi Yi Ke Da Xue Xue Bao J. West China Univ. Med Sci. Huaxi Yike Daxue Xuebao.

[40] Mahmoud M.E., Yakout A.A., Saad S.R., Osman M.M. (2015). Removal of potassium permanganate from water by modified carbonaceous materials. Desalination Water Treat..

[41] Virvadiya C., Kumari S., Choudhary V., Gupta V. (2014). Combined bio- and chemosorption of Mn(VII) from aqueous solution by PROSOPIS CINERARIA leaf powder. Eur. Chem. Bull.

[42] Chaudhary M. (2011). Use of Millet Husk as a Biosorbent for the Removal of chromium and Manganese Ions from the Aqueous Solutions. Int. J. Chem. Environ. Pharm. Res..

[43] Wan Ngah W.S., Hanafiah M.A.K.M. (2007). Removal of heavy metal ions from wastewater by chemically modified plant wastes as adsorbents: A review. Bioresour. Technol..

[44] Waranusantigula P., Pokethitiyook P., Kruatrachue M., Upatham E.S. (2003). Kinetics of cbasic dye (methylene blue) biosorption by giant duckweed (Spirodela polyrrhiza). Environ. Pollut..

[45] Mohan D., Pittman C.U. (2006). Activated carbons and low cost adsorbents for remediation of tri- and hexavalent chromium from water. J. Hazard. Mater..

[46] Varga M., Takács M., Záray G., Varga I. (2013). Comparative study of sorption kinetics and equilibrium of chromium (VI) on charcoals prepared from different low-cost materials. Microchem. J..

[47] Srinivasan K. (1986). Evaluation of Rice husk carbon for the removal of trace inorganic form water. Ph.D. Thesis.

[48] Iqbal M., Saeed A., Zafar S.I. (2007). Hybrid biosorbent: An innovative matrix to enhance the biosorption of Cd(II) from aqueous solution. J. Hazard. Mater..

[49] Malathi S., Srinivasan K., Gomathi M. (2015). Studies on the removal of Cr (VI) from aqueous solution by activated carbon developed from Cottonseed activated withsulphuric acid. Int. J. Chem. Tech. Res..

[50] Ansari R. (2006). Application of Polyaniline and its Composites for adsorption/Recovery of Chromium (VI) from Aqueous Solutions. Acta Chim. Slov..

[51] Jassal P.S., Raut V.P., Anand N. (2010). Removal of Chromium (VI) ions from Aqueous solution onto Chitosan and Cross-linked Chitosan Beads. Proc. Indian Natn. Sci. Acad..

[52] Marin N.M. (2022). Natural and Synthetic Polymers Modified with Acid Blue 113 for Removal of Cr^3+^, Zn^2+^ and Mn^2+^. Polymers.

[53] Gupta A., Jain R., Gupta D.C. (2015). Studies on uptake behavior of Hg (II) and Pb(II) by amine modified glycidyl methacrylate-styrene-N, N’-methylene bis-acrylamide ter- polyme. React. Funct. Polym..

[54] Dou X.B., Chai M.Y., Zhu Y., Yang W.T., Xu F.J. (2013). Aminated Poly(glycidyl methacrylate)s for Constructing Efficient Gene Carriers. ACS Appl. Mater. Interfaces.

[55] Wang L., Li F., Yao M., Qiu T., Jiang W., Fan L.-J. (2014). Atom transfer radical polymerization of glycidyl methacrylate followed by amination on the surface of monodispersed highly crosslinked polymer microspheres and the study of cation adsorption. React. Funct. Polym..

[56] Younis S.A., Ghobashy M.M., Samy M. (2017). Development of aminated poly(glycidyl methacrylate) nanosorbent by green gamma radiation for phenol and malathion contaminated wastewater treatment. J. Environ. Chem. Eng..

[57] Aversa T.M., da Silva C.M.F., da Rocha P.C.S., Lucas E.F. (2016). Influence of exchange group of modified glycidyl methacrylate polymer on phenol removal: A study by batch and continuous flow processes. J. Environ. Manag..

[58] Yu Y., Su J., Liu J., Li W. (2022). Magnetic Poly(glycidyl methacrylate) Microspheres with Grafted Polypyrrole Chains for the High-Capacity Adsorption of Congo Red Dye from Aqueous Solutions. Coatings.

[59] Chen W., Liu Y., Liu C. (2013). Preparation and use of magnetic poly(glycidyl methacrylate) resin in drinking water treatment. J. Appl. Polym. Sci..

[60] Cheng L., Wu J., Liang H., Yuan Q. (2020). Preparation of Poly(glycidyl methacrylate) (PGMA) and Amine Modified PGMA Adsorbents for Purification of Glucosinolates from Cruciferous Plants. Molecules.

[61] Benaglia M., Alberti A., Giorgini L., Magnoni F., Tozzi S. (2013). Poly(glycidyl methacrylate): A highly versatile polymeric building block for post-polymerization modifications. Polym. Chem..

[62] Waly A.I., Khedr M.A., Ali H.M., Ahmed I.M. (2022). Application of amino-functionalized cellulose-poly(glycidyl methacrylate) graft copolymer (AM-Cell-g-PGMA)adsorbent for dyes removal from wastewater. Clean. Eng. Technol..

[63] Mohy Eldin M.S., Elkady M.F., Abu-Saied M.A., Abdel Rahman A.M., Soliman E.A., Elzatahry A.A., Youssef M.E. (2010). Removal of cadmium ions from synthetic aqueous solutions using a novel nano-sulphonated poly glycidylmethacrylate cation exchanger: Kinetic and equilibrium studies. J. App. Poly Sci..

[64] Abu-Saied M., Fontana Nova E., Drioli E., Mohy Eldin M. (2013). Sulphonated poly (glycidyl methacrylate) grafted cellophane membranes: Novel application in polyelectrolyte membrane fuel cell (PEMFC). J. Polym. Res..

[65] Mohy Eldin M.S., Nassr A.A., Kashyout A.B.E., Hassan A. (2017). Novel Sulphonated Poly (Glycidyl Methacrylate) Grafted Nafion Membranes for Fuel Cell Applications. Polym. Bull..

[66] El-Aassar M.R., Tamer T.M., El-Sayed M.Y., Omer A.M., Althobaiti I.O., Youssef M.E., Alolaimi R.F., El-Agammy E.F., Alruwaili M.S., Mohy-Eldin M.S. (2022). Development of Azo Dye Immobilized Poly (Glycidyl Methacrylate-Co-Methyl Methacrylate) Polymers Composites as Novel Adsorbents for Water Treatment Applications: Methylene Blue-Polymers Composites. Polymers.

[67] Bulut Y., Aydin A. (2006). A kinetics and thermodynamics study of methylene blue adsorption on wheat shells. Desalination.

[68] Gode F., Pehlivan E. (2003). A comparative study of two chelating ion-exchange resins for the removal of chromium(III) from aqueous solution. J. Hazard. Mater..

[69] Gode G., Pehlivan E. (2005). Removal of Cr (III) ions by Turkish brown coals. Fuel Process Technol..

[70] Ho Y.-S. (2005). Effect of pH on lead removal from water using tree fern as the sorbent. Bioresour. Technol..

[71] Dubinin M.M., Zaverina E.D., Radushkevich L.V. (1947). Sorption, and structure of activated carbons, I. investigation of organic vapor removal. Zh Fiz Khim.

[72] Unlu N., Ersoz M. (2006). Removal characteristics of heavy metal ions onto a low-cost biopolymeric sorbents from aqueous solution. J. Hazard. Mater..

[73] Mohammad A., Rifaqat A.K.R., Rais A., Jameel A. (2000). Removal studies on *Citrus reticulata* (fruit peel of orange): Removal and recovery of Ni(II) from electroplating wastewater. J. Hazard. Mater..

[74] Ho Y.S., Porter J.F., McKay G. (2002). Equilibrium Isotherm Studies for the Sorption of Divalent Metal Ions onto Peat: Copper, Nickel and Lead Single Component Systems. Water Air Soil Pollut..

[75] Tan IA W., Ahmad A.L., Hameed B.H. (2008). Removal of basic dye using activated carbon prepared from oil palm shell: Batch and fixed bed studies. Desalination.

[76] Şeker A., Shahwan T., Eroğlu A.E., Yılmaz S., Demirel Z., Dalay M.C. (2008). Equilibrium, thermodynamic and kinetic studies for the biosorption of aqueous lead(II), cadmium(II) and nickel(II) ions on Spirulina platensis. J. Hazard. Mater..

[77] Helfferich F. (1962). Ion Exchange.

[78] Malik U., Hasany S., Subhani M. (2005). Sorptive potential of sunflower stem for Cr(III) ions from aqueous solutions and its kinetic and thermodynamic profile. Talanta.

[79] Smith J.M. (1981). Chemical Engineering Kinetics.

[80] Anari-Anaraki M., Nezamzadeh-Ejhieh A. (2015). Modification of clinoptilolite nanoparticles by a cationic surfactant and dithizone for removal of Pb(II) from aqueous solution. J. ColloidInterf. Sci..

[81] Heidari-Chaleshtori M., Nezamzadeh-Ejhieh A. (2015). Modified clinoptilolite nano-particles with Aspartic acid for removal of Cu(II) from aqueous solutions: Isotherms and kinetic aspects. New J. Chem..

[82] Hameeda B.H., China L.H., Rengarajb S. (2008). Removal of 4-chlorophenol onto activated carbon prepared from rattan sawdust. Desalination.

[83] Temkin M.I., Pyzhev V. (1940). Kinetics of ammonia synthesis on promoted iron catalysts. Acta Physicochim..

[84] Langergren S., Svenska B.K. (1898). Zur theorie der sogenannten adsorption geloester. Stoffe Veternskapsakad Handl..

[85] Ho Y.S., Mckay G. (1998). The kinetics of sorption of basic dyes from aqueous solution by sphagnum moss peat. Can. J. Chem. Eng..

[86] Ozacar M., Sengil I.A. (2005). A kinetic study of metal complex dye sorption onto pinedust. Proc. Biochem..

[87] Ho Y.S., McKay G. (1999). Pseudo-second order model for sorption processes. Proc. Biochem..

[88] Tseng R.L. (2006). Mesopore control of high surface area NaOH-activated carbon. J. Coll. Interf. Sci..

[89] Khan T.A., Dahiya S., Ali I. (2012). Use of kaolinite as adsorbent: Equilibrium, dynamics and thermodynamic studies on the adsorption of Rhodamine B from aqueous solution. Appl. Clay Sci..

[90] Zhao G., Li J., Wang X. (2011). Kinetic and thermodynamic study of 1-naphthol adsorption from aqueous solution to sulfonated graphene nanosheets. Chem. Eng. J..

[91] Alkan M., Demirbaş Ö., Çelikçapa S., Doğan M. (2004). Sorption of acid red 57 from aqueous solution onto sepiolite. J. Hazard. Mater..

[92] Kifuani K.M., Mayeko AK K., Lopaka B.I., Bokolombe P.N., Ondongo T.M., Bakambo G.E., Lunguya J.M. (2018). Kinetic and thermodynamic studies adsorption of Methylene Blue (MB) in aqueous solution on a bioadsorbent from Cu-cumeropsis mannii Naudin waste seeds. Int. J. Biol. Chem. Sci..

[93] Wu J., Xia A., Chen C., Feng L., Su X., Wang X. (2019). Adsorption Thermodynamics and Dynamics of Three Typical Dyes onto Bio-adsorbent Spent Substrate of Pleurotus eryngii. Int. J. Environ. Res. Public Health.

[94] Yagub M.T., Sen T.K., Ang M. (2014). Removal of cationic dye methylene blue (MB) from aqueous solution by ground raw and base modified pine cone powder. Environ. Earth Sci..

[95] Eldin M.S.M., Gouda M., Youssef M.E., El-Shazly Y.M.S., Farag H.A. (2016). Removal of methylene blue by amidoxime polyacrylonitrile-grafted cotton fabrics: Kinetic, equilibrium, and simulation studies. Fibers Polym..

